# Expanding the disaster risk management framework: Measuring the constructed level of national identity as a factor of political risk

**DOI:** 10.4102/jamba.v8i1.232

**Published:** 2016-08-31

**Authors:** Barend Prinsloo, Gerrit van der Waldt

**Affiliations:** 1 Security Studies and Management, North-West University, South Africa; 2 Public Governance, North-West University, South Africa

## Abstract

Political risk is identified as a dominant risk category of disaster risk management (DRM) which could negatively affect the success of those measures implemented to reduce disaster risk. Key to political risk is the construct of national identity which, if poorly constructed, could greatly contribute to political risk. This article proposed a tool to measure the construct of national identity and to provide recommendations to strengthen the construct in order to mitigate the exacerbating influence it may have on political risk and ultimately on DRM. The design of the measurement tool consisted of a mixed methodological approach employing both quantitative and qualitative data. The data collection instruments included a literature review (which is shortly provided in the previous sections) and an empirical study that utilised data obtained through structured questionnaires. Although the results of the proposed measuring instrument did not include a representative sample of all the cultures in South Africa, the results alluded to different levels for the construction of national identity among black and white respondents, possibly because of different ideological expectations among these groups. The results of the study should be considered as a validation of the measuring tool and not necessarily of the construct of national identity in South Africa. The measuring tool is thus promising for future studies to reduce political risk and ultimately disaster risk.

## Introduction

Disaster risk management (DRM) is a multi-faceted, interdisciplinary field. DRM in terms of the *South African Disaster Management Act* (57 of 2002) refers to the continuous and integrated multi-sectoral, multidisciplinary process of planning and implementing of measures aimed at preventing or reducing the risk of disasters, mitigating the severity or consequences of disasters, ensuring emergency preparedness, prompting a rapid and effective response to disasters and facilitating post-disaster recovery and rehabilitation (South Africa 2002). DRM, as a young and highly dynamic field of study, is influenced by a wide variety of factors including economic, technological, geographical, environmental and political issues, trends and conditions (Wisner, Gaillard & Kelman 2012:xxviii). This emerging discipline finds itself at the boundaries of a wide variety of natural and social sciences disciplines including geography, spatial planning, development studies, political sciences, sociology, public management and communication studies (Van der Waldt 2009). This highlights the ‘emerging cross-cutting’ nature of DRM (Holloway 2009:104). In the case of South Africa, Van der Waldt conducted a survey and by means of content analysis identified key foci of DRM as portrayed by qualifications, programmes, research reports and the United States Agency for International Development (USAID) knowledge products (Van der Waldt 2013). The nine categories in Table 1 (Management, Development, Communication and ICT, Health and Safety, Environment, Economy and Financial aspects, Education and Training, DRM theory, and Government, Governance and Politics) were identified.

**TABLE 1:** Knowledge categories of disaster risk management.

Knowledge category	Study foci
Management	Planning, risks (including hazards, risk assessments, vulnerability); establish and manage disaster management structures; disaster response and recovery; resource utilisation; etc.
Development (social dimension)	Community dynamics and demographics; communities at risk; indigenous knowledge base; sustainable livelihood assessments; building resilience and enhancing livelihoods; etc.
Communication and ICT	Crisis communication; early warning systems; forecasting; etc.
Health and safety	Nutrition; epidemics; diseases; unsafe living conditions and practices; etc.
Environment	Climate change; weather patterns; agriculture; natural resources; etc.
Economy and financial aspects	Economic conditions; financial risks; costs of disasters; etc.
Education and training	Skills development; institutional people capacity; etc.
DRM theory	Theory and theory development; model building; etc.
Government, governance and politics	Statutory and regulatory frameworks; guidelines for operational organisational structures and arrangements; civil defence agencies; institutional resource allocation; disasters and political dynamics; pre-disaster risk reduction and post-disaster recovery; international policy, aid and relief; international treaties and protocols; geopolitical risks; etc.

*Source*: Adapted from Van der Waldt, G., 2013, ‘Disaster risk management: Disciplinary status and prospects for a unifying theory’, *Jàmbá. Journal of the African Centre for Disaster Studies* 5(2), 13. http://dx.doi.org/10.4102/jamba.v5i2.76

ICT, Information and Communication Technology; DRM, disaster risk management.

This categorisation system illustrates the multidimensional and multidisciplinary nature of DRM. It is further evident that elements of risk feature prominently in virtually all categories. For the purposes of this article, however, it should be noted that political risk is regarded as a dominant category. The World Economic Forum’s Global Risks Report categorises risks into economic, environmental, societal, technological and political dimensions (Bekefi & Epstein 2006:14; WEF 2014). The focus of this article rests in this political space. The main premise of this article is that disaster management efforts will be less effective in a country with high levels of political risk. The inadequate construction of national identity is seen as pivotal to exacerbate political risk.

Political risk is a rather broad, multidimensional concept which requires conceptual and contextual clarification. The conceptual context of South Africa’s constitutional democracy remains fluid and is a highly politically contested arena. In its broadest sense, political risk can be defined as the potential negative consequences arising from political dynamics and social behaviour (Cawthra & Luckman 2003:74). Political dynamics and behaviour are intertwined with power relations and dominant ideology of society (Bekefi & Epstein 2006:14; Ghadar, Kobrin & Moran 1983). The foundational elements associated with this include levels of authority and legitimacy, political culture and national identity (Howell 1994:6; Mckellar 2010). Political risk affects entire societies, especially social relations (Mckellar 2010:7). Society is affected by the laws of the state, and social dynamics are guided by a framework ultimately set by political authority and social relations. The political risk can further be categorised into broader macro or international (geo-)political risks, country-specific risks, sector or industry risks, and more micro-level organisational risks (Bekefi & Epstein 2006:15). Country-specific risks typically emerge from an unstable socio-political situation within the country. It is further postulated that the distinction between social and political risks is often blurred (Bekefi & Epstein 2006:15). South Africa is a developing country, and given the country’s political dynamics over many years, political risk has always been an issue and will remain important in the evolution of the future political landscape in South Africa. The principles associated with DRM provide a useful framework to assess threats, hazards, vulnerabilities and mitigation strategies in the South African political landscape. Such threats and vulnerability assessments should lead to specific likelihood and impact assessments of identified issues. As such, political risk is linked to the broader framework of DRM.

The main contributions of this article are centred on the following objectives:

- To accentuate the significance of political risk in a disaster risk framework.
- To illustrate the significance of national identity as key determinant within political risks.
- To categorise the various factors in national identify by means of an ideological frame (towards a typology).
- To design a measuring instrument (BP Model) to gauge the level of national identity in a country.
- To provide an empirical methodological example of the application of this measuring instrument.

## ‘National identify’: A conceptual clarification

One school of thought maintains that ‘national identity’ is actually a ‘four-in-one’ combination of institutional identity, interest identity, cultural identity and non-national community identity, with formative mechanisms characterised by the unity of the primordial state, the constructive, expressive forms characterised by the unity of consciousness and action, content characterised by the unity of politics and culture and maintenance mechanisms characterised by the unity of emotion and self-interest (Zhuojun & Hualing 2014:139). Another school of thought does not focus so much on what national identities means but on how it is constructed in the first place. This latter school maintains that national identities need not be based either on ethnicity or abstract principles but can be created, sustained and altered through a political process. Such an identity can change over time (Spinner-Halev 2008:622). The construction of national identity takes place in at least two ways.

### Deliberate construction

New national identities are constructed throughout the world. It was for example constructed in post-apartheid South Africa and in post-communist states. There are similarities in efforts to build new national identities in post-apartheid South Africa and in post-communist states which are useful to illustrate this point. The presumption that the national identities of post-communist states were expected to provide the foundations for statehood and promote their ability to persist as independent states (Melnykovska, Schweickert & Kostiuchenko 2011:1056) would probably be equally true in the case of post-1994 South Africa (Prinsloo 2016:85). In the same way that the post-communist states had to rediscover their ‘national self’ and re-define the ‘others’ by stressing the similarities of the in-group and its differences with those outside the political community (Melnykovska *et al*. 2011:1056), South Africa’s ‘national self’ was reviewed and redefined during the pre-election negotiations and captured in its constitution (Prinsloo 2016). Processes such as these could, however, be detrimental to the building of a multicultural society or pluralist political setting in a state because when a country declares itself a ‘national state’, it usually leads to an ethnic definition of ‘nationality’ (Korostelina 2003:141–142). Within the ethnic concept of national identity, some people perceive their nation as being built around a core ethnic community into which ethnic minorities should assimilate. They see their nation as a monoethnic and monolingual one (Korostelina 2008:208). As such, the manner in which the construction of a state and the reshaping of national identities take place and the ideological basis it presents are essential elements to move from totalitarianism to political pluralism. It would not be possible to do so without considering the numerous dimensions and parameters of national identity, namely political, territorial, ecological, ethnic, social and religious senses of such values as ‘survival’, ‘prosperity’, ‘equality’, ‘freedom’, ‘development’ and ‘justice’ and understanding of national interests, priorities and goals (Korostelina 2003:141–142).

### Personal acceptance of a greater sense of identity

Through the process of building new national identities, it could be argued that nations are imagined, in that they are generally the result of shared perceptions among people who think of themselves as being part of that nation (i.e. community). Because most people in a nation will never see, let alone meet, one another, their bond is a social construction; one in which communication has an important role. Indeed, much of the scholarship on national identity has emphasised the importance of communication in civic engagement and social construction (Coe & Neumann 2011:141). More broadly seen, national identity refers to how societies relate to their own unique characteristics and is based on Huntington’s four elements of civilisation: religion, history, customs and social institutions. The principal components of national identity include four dimensions: belief structure, national heritage, cultural homogeneity and consumer ethnocentrism (Thelen & Honeycutt 2004:59). Shared perceptions of people build on the ideal of the ‘nation’ being multicultural, with equal rights for all ethnic groups and even some elements of autonomy and self-governance. They see their state as a society within which ethnic minorities should be guaranteed resources to maintain their ethnic culture and communities. In order for this to work, the different ethnic groups must have an opportunity to receive education in their language, and their cultural heritage must be part of the country’s heritage (Korostelina 2008:208). National identity can in such cases be regarded as a person’s psychological affiliation to his or her country of residence (Fuller-Rowell, Ong & Phinney 2013:406) based on the idea that all people and cultures are equal. A union of personal and national identity is likely a prerequisite for national self-identification (Barnes *et al*. 2014:638). The personal acceptance of the greater sense of identity could be upheld through a civil contract between the people and the state about rights and obligations. In such cases, the constitution, rule of law and civic responsibility as the main features of the nation will be more significant than ethnicity. Such a view will allow the nation to be built on a distinctive, non-ethnic civic culture into which all citizens should be integrated (Korostelina 2008:208).

It is thus clear that the state has an important role to play in the construction of national identity, and it should not be dominated by individual identity or ethnical groups. Furthermore, these two ways indicate that the individual part of a certain ethnic group is either provided with an external constructed sense of national identity or takes part in the shaping of national identity. In both cases, the vehicle to shape national identity remains the state through political processes. In summary, the basic meaning of ‘identity’ is to establish a sense of belonging, or the need to rediscover a sense of security and a sense of collectivity in a plural setting (Prinsloo 2016). It should be understood that the senses of identity comes initially from the individual but its widespread recognition generally comes through the vehicle of the state, and it eventually ends at the political level (Prinsloo 2016:84). Importantly, any doubts shown by members of the polity about the rationality of the system could weaken the legitimacy of the political identity and could inflict harm on the political system (Xiaomei & Shimin 2014:162–163). As an important backdrop to measure the construction of national identity, an overview of the extent to which these issues remained on the South African political agenda since 1994 is provided next.

### The indicators of national identity

For purposes of constructing a measuring instrument to gauge the level or quality of national identity, it was necessary to establish the key indicators associated with it. For this purpose, an extensive literature survey was undertaken to uncover the core determinants as highlighted by a scholarly discourse on the topic provided earlier. The principles of content analysis were then utilised to group or classify related indicators of national identity in both ways of its construction (i.e. ‘deliberate construction’ and ‘personal acceptance of a greater sense of identity’). The results of this endeavour revealed 32 indicators (italicised in the list) which were identified in an effort to ring-fence or demarcate national identity in all its dimensions and elements (which are referred to as the ’indicators of national identity’):

1. A *sense of justice* that prevails throughout society (Gelisli & Beisenbayeva 2015:487).
2. The *rule of law* prevails and is applied in an unbiased fashion (Molina, Phillips & Sidanius 2015:226).
3. The *constitution is respected* by the citizenry and all organs of state (Korostelina 2003:141–142).
4. The *civic rights* of all citizens are respected and protected through legislation (Grant 2016:62).
5. Different *belief structures* are respected and protected (religious and others) (Korostelina 2003:141–142).
6. Measures and legislation are in place to safeguard the *equality of all cultures* (Molina *et al*. 2015:225).
7. *Political freedom* is ensured (Korostelina 2003:141–142).
8. The prevailing sense of *national identity is accepted* by all members of society (Zeugner-Roth, Žabkar & Diamantopoulos 2015:27).
9. The sense and concept of *national identity is allowed to evolve and change* (Molina *et al*. 2015:225).
10. The state is playing an active role in the creation and protection of national identity (*state-created identity*) (Gelisli & Beisenbayeva 2015:487).
11. *Communication* between different ethnic groups exists (Coe & Neumann 2011:141).
12. A *unique national identity* is allowed to develop independent of ethnic identities (Korostelina 2008:208).
13. *Ethnic equality* prevails in the face of different cultures and a unique national identity (Molina *et al*. 2015:226).
14. *Cultural homogeneity* is enhanced through a common sense of national identity (Molina *et al*. 2015:226).
15. Common *national interests, priorities and goals* are enshrined within the concept of a national identity (Grant 2016:59).
16. *Nationalism* is enhanced through the prevailing concept of national identity (Grant 2016:59).
17. People of all ethnicities have a common understanding of *citizenship* (Grant 2016:59).
18. People of different ethnicities respect other cultures as part of the overarching national identity (*Autonomy*) (Grant 2016:59).
19. *Patriotism* is enhanced through the prevailing concept of national identity (Ha & Jang 2015:473).
20. *Territorial affinity* is enhanced through the prevailing concept of national identity (Gelisli & Beisenbayeva 2015:487).
21. *Social identification* is enhanced through the prevailing concept of national identity (Zeugner-Roth *et al*. 2015:27).
22. There is evidence of a union of personal and national identity (Barnes *et al*. 2014:638).
23. *Psychological affiliation* to the prevailing concept of national identity is strong (Fuller-Rowell *et al*. 2013:406).
24. There is evidence of growing *assimilation* of ethnical identities and the concept of national identity (Ha & Jang 2015:473).
25. The sense of national identity drives *common cause development* (Korostelina 2008:208).
26. The *national heritage* of the country is protected as a result of a strong national identity (Gelisli & Beisenbayeva 2015:487).
27. *Ecological preservation* takes place as a result of a strong national identity (Korostelina 2003:141–142).
28. The concept of national identity includes the notion to allow prosperity for all people (Korostelina 2008:208).
29. *Consumer ethnocentrism* is enhanced through the prevailing concept of national identity (Zeugner-Roth *et al*. 2015:26).
30. *Group solidarity* is enhanced through the prevailing concept of national identity (Molina *et al*. 2015:225).
31. *Sectarianism* is reduced through the prevailing concept of national identity (Goble 2016:37–38).
32. Citizens do not feel discriminated and persecuted owing to their ethnicity (*Feelings of survival*) (Grant 2016:62).

### Ideological conceptualisation of national identity and its indicators

**FIGURE 1:**
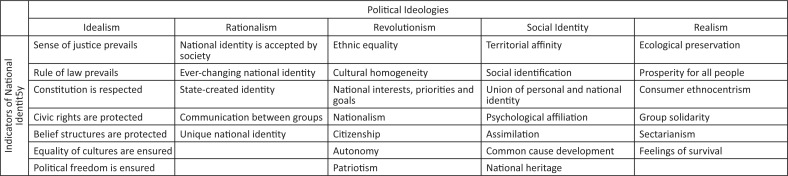
Ideological conceptualisation of national identity and its 32 indicators.

To further conceptualise national identity, the 32 indicators were categorised and grouped according to ideological origins to determine, as one outcome of the study, if any possible correlation existed between political ideological expectations as part of the national identity concept and different ethnic groups. The political ideologies were idealism, rationalism, revolutionism, social identity theory and realism (see Figure 1). Short interpretations of the five political ideologies followed in this article are:

- **Idealism:** Reality as a mental construct should in some sense correlate with the appearance of the object (Schulting & Verburgt 2011:viii). Therefore, for the idealist, national identity as a construct must bear some sort of evidence to its conceptual expectations.
- **Rationalism:** The rationalist’s view of the state of nature is one of mutual cooperation (as opposed to the realist’s view of continual competition and conflict) (Labuschagne 2000:7). In this case, proponents of a rational view of national identity would have a more fluid expectation and accepting attitude towards other people’s (differing) viewpoints.
- **Revolutionism:** The acceptance of a world divided into camps in terms of acceptance or rejection of a given ideology; arising from this, an insistence on ideological homogeneity (Labuschagne 2000:7). Thus, it is expected by revolutionists that national identity should be perpetually redefined while competing viewpoints on national identity should be rejected or incorporated into the revolutionary ideological framework.
- **Social identity:** Social identity theory posits that the social categories individuals fall within provide insights into how those individuals define themselves. Identification is the process by which individuals come to define themselves in terms of a perceived social group or category (Cannella Jr, Jones & Withers 2015:438). Within this view, people would be influenced by external loci or elements from their social category which they choose to accept to shape their perceptions of national identity.
- **Realism:** Realism is identified by a pessimistic view of human nature that is obsessed with power for selfish reasons (Labuschagne 2000:6). Realist proponents of national identity would favour elements that ensure the protection of their selfish interests or survival.

## Political risk analysis of South Africa

Political risks are subjected to a risk assessment index of political, economic and social conditions prevalent in a country. Recent political risk assessments of South Africa, for example, reveal high levels of corruption, social tension and regulatory uncertainty. The global risk management company, Aon Risk Solutions, in its *Terrorism and Political Violence Map*, rates South Africa at a medium score of 3 in terms of political risk and a low score of 2 for the threat of terrorist attacks (Solutions 2014). Political risk assessments are however much broader in scope than mere violence dimensions and therefore scholars such as (Fouche 2003; Howell & Chadwick 1994) Venter utilises categorisation systems in attempts to design a typology of political risks (Venter 2005). A points-based scoring system to analyse political risks, including the following variables, was designed (Howell & Chadwick 1994):

- Bad neighbours (3 points): the regional situation the country finds itself in.
- Authoritarianism (7 points): the lack of democracy.
- Staleness (5 points): when a leader is in power for more than 10 years.
- Illegitimacy (9 points): an uncoerced and positive acceptance of the state by the citizenry; the gap between acceptability and the government’s persistence in power could pose a high political risk.
- Generals in power (6 points): in the face of instability or the absence of a competent civilian government, the military authorities often step in to take control.
- War or armed insurrection (20 points): war destroys physical facilities, disrupts the economy and brings about losses in a number of other ways.

Venter expanded on this typology and in turn employed 13 risk indicators to determine the current political risk profile of South Africa (Fouche 2003; Venter 2005). Due to the completeness of this categorisation system, it will be utilised to briefly elucidate South Africa’s political risk profile (Table 2). An in-depth political risk analysis of South Africa falls outside the scope of this article, but it is necessary for purposes of contextualising the issue of national identity. Cognisance of political risk factors is also imperative to gauge the potential significance thereof in a disaster risk reduction frame.

**TABLE 2:** Risk indicators and South Africa’s political risk profile.

Risk indicator	Risk rating (1–5; 5 = high risk)	Supporting argument(s)
Threatening neighbouring states	1	South Africa has focused its foreign policy on promoting the integration of the SADC, the unity and the renewal of the African continent. South Africa is also committed to work with neighbouring countries for a stable geopolitical system of governance. Considering South Africa’s political risk profile, it can be concluded that South Africa faces no immediate threat by the so-called ‘bad’ neighbours.
Authoritarian measures to retain power	3	Authoritarian measures in South Africa do not seem to amount to high political risk *per se*, but indications are that the ruling ANC policies and practices do not instil confidence in South Africa’s state institutions.
Staleness of incumbency and calibre of leadership	3	Staleness typically occurs when a leader has been in power for a period of longer than 10 years. Recent national and international commentators seem to agree that serious question marks exist as far as the quality of South Africa’s political leadership is concerned. Controversies such as ‘Guptagate’, Nkandla, the ‘Spy’-tapes and the various (yet untested) corruption charges against President Zuma are particularly instructive and significant.
Legitimacy of government	3	Although there is little doubt that the ANC enjoyed legitimacy as a government since 1994, it faces some challenges, especially at the local sphere of government. Research conducted by the Southern African Peace and Security Studies clearly reflects relative high levels of dissatisfaction with municipalities on a broad basis. Low levels of public confidence both in local government and trust in elected leadership and public officials are reason for concern, and increasingly violent and destructive ‘service delivery’ protests may spiral out of control and pose significant political risks in the foreseeable future.
Military involvement in politics	2	Political interference by the SANDF does not pose a political risk, but of concern in the broader political context is the issue of the operational readiness of the military since its budget has been stripped to the bone, resulting in capability gaps and poor maintenance of sophisticated equipment. Of further concern are assertions that the intelligence services remain central to the power structure of the South African state.
Extremism, religious tension and terrorism	1	South Africa does not have a situation where religious freedom is suppressed or where civil law is replaced by religious law. South Africa also does not experience a surge in radicalism. Concerns that Islamist movements such as al-Qaida and ISIS have infiltrated the country are relatively low and right-wing militancy also seem to have declined significantly.
Socio-economic conditions	3	Socio-economic conditions measure the satisfaction or dissatisfaction with the socio-economic policies of the government in a country. Relevant factors include infant mortality, medical care, interest rates, disparities between different strata of society, unequal distribution of wealth, crime, unemployment, illiteracy, drug use and health conditions. Commentators especially highlight the extreme differences in wealth, youth unemployment and the Aids pandemic as high-risk factors in South Africa. A positive aspect, however, is the rapid growth in the middle class, which promises greater longer term political stability and economic growth.
Safety and security (law and order)	4	South Africa experiences exceptional high levels of crime and corruption. This is cited as some of the biggest concerns for foreign and local investors in South Africa. Of further concern is the recent spate of xenophobic attacks against foreigners, which do pose a threat to security on a national scale.
Trade union activism and labour policy	2	The tripartite alliance between Cosatu and the ruling ANC government is characterised by serious tension. Violent strikes are a common feature of the industry. South Africa has a long history of labour unrest, and internationally a perception exists that the labour law system only favours workers. Labour productivity is generally low. According to the 2014 World Competitiveness Report, South Africa dropped from the previous year and ranks 113th for labour market efficiency; 143rd for its rigid hiring-and-firing practices; 140th for the lack of inflexibility in wage determination by companies; and 144th for significance of tensions in labour relations.
Administrative (in)competence of government	4	Cadre deployment, affirmative action and nepotism are reasons cited for low competency levels in the South African Government. As implementers of national legislation, the capacity of government institutions is severely compromised by unresponsive and unskilled officials.
Macro-political and economic circumstances	3	Aspects such as income tax, structural problems in the economy (e.g. schooled labour and trained human capital and labour productivity), macroeconomic indicators and the ability to attract foreign direct investment are all indicative of potential political threats. According to the International Monetary Fund’s 2014 report, South Africa has a stable and resilient economy but the decline in economic growth, low creditworthiness, growing national debt, the huge public sector wage bill and limited labour-intensive growth are cited as reasons for concern.
The security of property and the discourse on nationalisation	2	Agricultural land reform and the possible nationalisation of mines and other assets raised fears in general about the security of private property. These issues remain highly sensitive and somewhat uncertain.
Racial, ethnic and language cleavag-es (national identity)	3	Racial and ethnic tension generally represents a negative political climate. The exclusion of minority groups from political and social structures is a serious cause for concern. This exclusion causes people to become alienated, loose their sense of belonging and this jeopardises cohesion in society. South Africa has a long history of racial and ethnic divisions characterised by substantial exclusivism. These differences still lie deep and remain a potential source of tension. It is further evident that support for political parties is still largely racially based. Commentators concur that South Africa remains a deeply divided society. The SARB survey (2013), conducted by the Institute for IJR, reflects that limited progress has been made in areas such as reconciliation, social cohesion and nation-building in recent years (Institute for Justice and Reconciliation 2013). Analysts agree, however, that the majority of citizens continue to support the ideal of national unity, despite strong associations with other identity groups based on language, ethnicity and race. Thus, in terms of political risk, this probably points towards the need for new consideration of a more inclusive and tolerant national identity.

*Source:* Fouche, P.J., 2003, ‘A political-security risk analysis of Uganda’, Unpublished Masters Dissertation in Security Studies, University of Pretoria, Pretoria and Venter, A., 2005, ‘A comment on political risks for South Africa’, *Strategic Review for Southern Africa* XXXII(1), 28–54

SADC, Southern African Development Community; ANC, African National Congress; SANDF, South African National Defence Force; SARB, South Africa Reconciliation Barometer; IJR, Institute for Justice and Reconciliation.

Based on the above brief analysis, it seems that South Africa probably remains in the medium risk category. One of the core dimensions of political risk is national identity (Fouche 2003; Howell & Chadwick 1994; Venter 2005). It is thus evident that issues of social cohesion and national identity should always remain high on the state’s political agenda.

### Efforts to build national identity in South Africa since 1994

South Africa’s transition to democracy from 1994 onwards was characterised by numerous attempts to deracialise and bring together a diverse society subjected to forced segregation and racial antagonism. By the time that Mbeki was recalled as president by the ANC in 2008, indications were that the government had already failed in its efforts to develop national unity through the philosophy of the African Renaissance and the values of *ubuntu.*^1^ By the time that President Jacob Zuma came to power in 2009, it would be fair to say that an inclusive national political identity was not created and South Africa’s national identity was in a fragmented state (Prinsloo 2016:85).

President Jacob Zuma became the president of South Africa in 2009 being at the helm of what was considered to be the ‘first phase’ of democratisation (which meant politically and constitutionally the abolishment of a state form associated with white minority rule and the introduction of major redistributive programmes [SACP 2014]) (Prinsloo 2016).

Apart from any ideological or party strategies to be implemented, two events demanded a volte-face from the continuation of the policies of his predecessors (Prinsloo 2016:87). The first event was the massacre in Marikana on 16 August 2012 when the Police fired on a group of striking miners, killing 34 and injuring 78 others. Apart from indicating the potential for uncontrolled state violence, it also pointed out underlying social ills allegedly perpetuated by Black Economic Empowerment (BEE) and the widening income equality in South Africa (Guha 2013:7) (Prinsloo 2016). The second event which demanded a volte-face of government policies was related to the formation of the Economic Freedom Fighters (EFF) as a political party. It is important to reflect on the EFF’s policies as they provide an insight into the yearnings of a new generation who have not benefitted as much politically and economically as older members of the ANC and have little memory of the struggle against apartheid (PRS_Group 2014:U4) (Prinsloo 2016). The EFF demanded radical transformation of the South African society, including the expropriation of land without compensation and the nationalisation of mines, banks and other strategic sectors of the economy – also without compensation (EFF 2014). Here, it should be noted that a civic state that simply inculcated a sense of belonging that was detached from any group would too readily lead to forgetting as well; it might also lead to viewing the state strictly in economic terms (Spinner-Halev 2008:620). The important point here is that the state has a responsibility to help form the national identity of a society which is inclusive of all groups and should not be used as merely the vehicle to transform or maintain certain economic conditions in society. However, the perception of a lack of (economic) transformation in the South African society combined with the failure of the government to successfully transfer the concept of an inclusive national identity to the next generation only increased South Africa’s political risk profile. It should be noted that even if the state fails to adequately construct national identity, it should not be discarded. Indeed, the separation of identity from politics can be a ‘dangerous desire’, since it may severe collective memory from the territory of the state, and leave it nearly completely in the hands of individual identity groups. Collective memories place people in a narrative, one that reaches from the past to the present and into the future. These memories are important because identities are mainly shaped through individual and collective memories (Spinner-Halev 2008:620).

## An instrument to measure the construct of national identity

The main premise of this article is that disaster management efforts will be less effective in a country with high levels of political risk. The inadequate construction of national identity is seen as pivotal in exacerbating political risk. As such, it would be highly useful to be able to measure the construction of national identity in a country to determine its impact on political risk. To do this, the design of the measurement tool rested on a few assumptions:

- From the literature review in the previous sections, it was determined that national identity is constructed through the state either by direct participation of interest groups, or it is provided as a preconceived construct to interest groups.
- The most important point is, however, that in both cases the construction of national identity would not be successful if the individual or different interest groups do not accept the proposed identity.
- The proposed identity is multi-faceted and consists of numerous indicators which must be considered.

### Design of the instrument to measure the construction of national identity

The design of the measurement tool consisted of a mixed methodological approach employing both quantitative and qualitative data. The data collection instruments included a literature review (which is shortly provided in the previous sections) and an empirical study that utilised data obtained through structured questionnaires. Once the data were obtained, the results of the empirical study were statistically analysed and compared with the conclusions and assumptions from the literature review.

The empirical study was conducted as follows:

- A structured questionnaire was developed which contained 32 statements based on the 32 indicators of national identity. The 32 statements were structured to capture participants’ perceptions on the degree of achievement (i.e. level of prevalence) of these indicators of national identity in South Africa.
- Participants could rate how strongly they agreed with the statements on a 5-point Likert-scale (1 = strongly disagree, 2 = ‘disagree’, 3 = ‘neutral’, 4 = ‘agree’ and 5 = ‘strongly agree’).
- In the validation phase of the research, two cultural groups were chosen to partake in the Potchefstroom area in the North-West Province of South Africa, namely white Afrikaans and English-speaking people (*n* = 8) and black residents (*n* = 56).
- The responses obtained from this trail group were then statistically analysed, compared and depicted through spidergrams.

The aim of the measuring instrument was to ensure that it accurately captured the levels of development of the indicators of national identity in any state environment and not only be restricted to South Africa. It should therefore be noted that although the results of the trail phase centred on a limited geographical area in South Africa, the approach followed in the research could be adapted to incorporate geographical area or country of any size. Also, the results of the study should be considered as a validation of the measuring tool and not necessarily of the construct of national identity in South Africa. To adequately measure the construct of national identity in South Africa, the empirical study needs to be expanded:

- increasing the number of respondents
- including all ethnic groups represented in South Africa
- including feedback from respondents located throughout South Africa and not only one geographical location.

### Results of the validation phase

**FIGURE 2:**
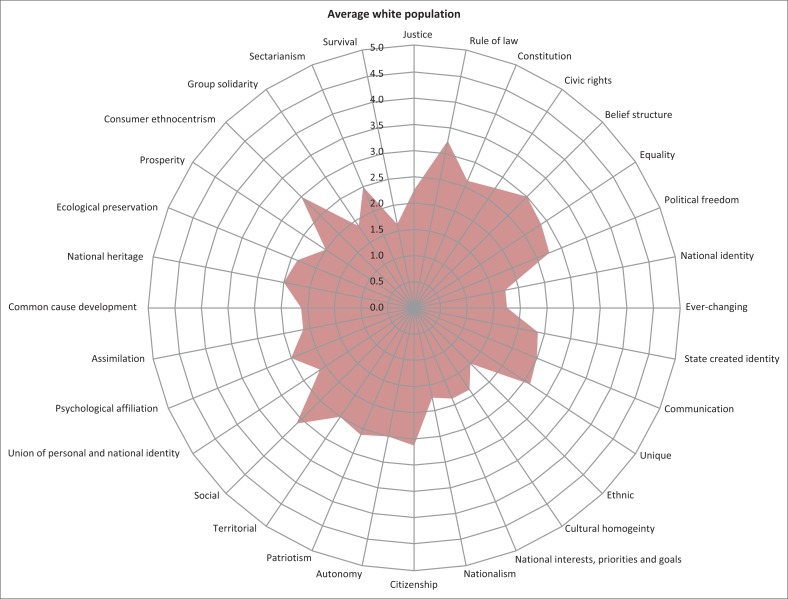
Spidergram depicting the extent of the indicators of national identity (white respondents).

**FIGURE 3:**
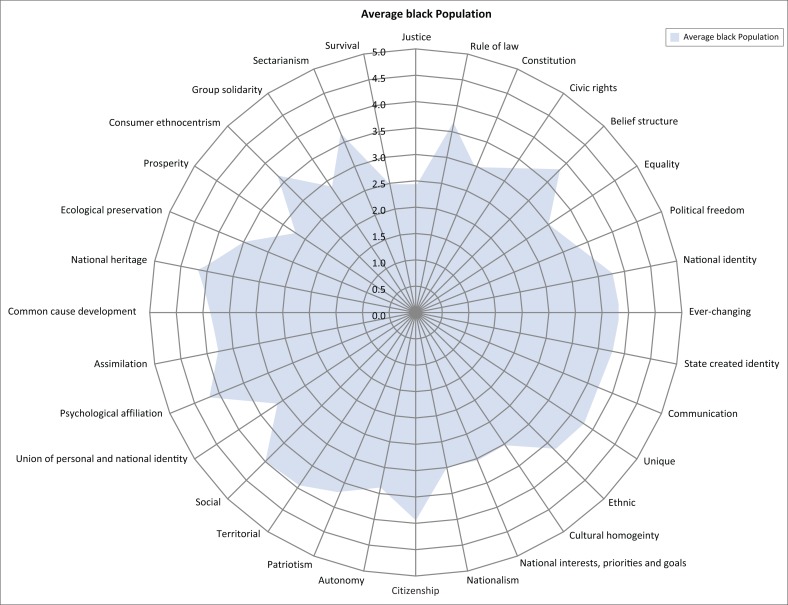
Spidergram depicting the extent of the indicators of national identity (black respondents).

**FIGURE 4:**
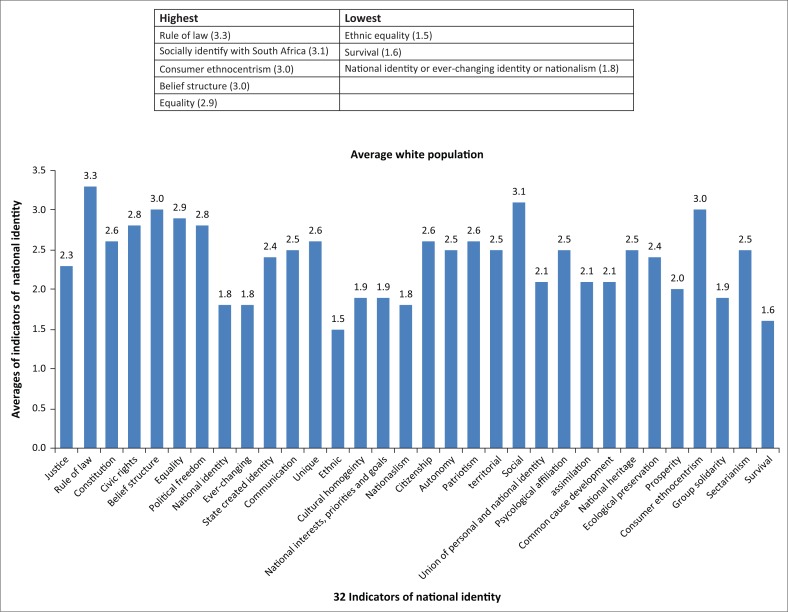
Highest and lowest averages for the indicators of national identity (white respondents).

**FIGURE 5:**
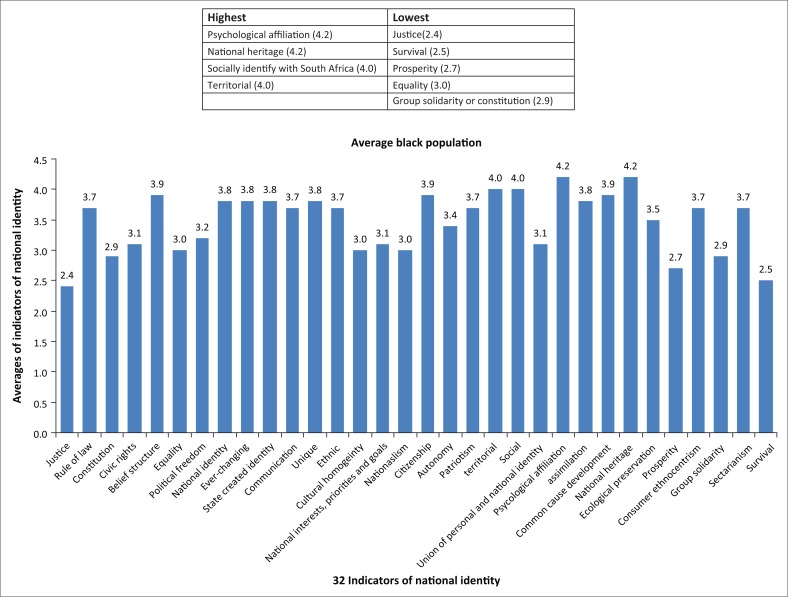
Highest and lowest averages for the indicators of national identity (black respondents).

The spidergram for the white Afrikaans and English-speaking people (*n* = 8) appeared as shown in Figure 2. The spidergram for the black residents (*n* = 56) appeared as shown in Figure 3. The results show the highest and lowest scoring indicators of national identity for the white respondents as shown in Figure 4. The results show the highest and lowest scoring indicators of national identity for the black respondents as shown in Figure 5.

The 32 indicators of national identity were incorporated into five political ideologies (see Table 2). This allowed the identification of not only those indicators of national identity which were the least or most developed among the two ethnic groups, but it also allowed for the comparison of the different political ideological approaches that were seemingly important to the different ethnic groups. This was significant as it pointed to possible contradicting expectations from different ethnic groups to construct national identity and also the different approaches the state should consider incorporating to successfully construct national identity. The following became evident from the results of the validation phase:

- Judged by the high and low scores of the two categories of respondents, and as depicted in the spidergrams, the development of national identity is seemingly less pronounced among white people and more pronounced among black people.
- White people’s strongest affinity to indicators of national identity were within the idealism category (rule of law, belief structure, equality).
- White people’s weakest affinity to indicators of national identity was within the revolutionism (ethnic supremacy, nationalism) and rationalism categories (national identity is [not] accepted, ever-changing identity).
- Black people’s strongest affinity to indicators of national identity was within the social identity categories (socially identifying with South Africa, psychological affiliation with South Africa and strong feelings towards national heritage, territorial affinity).
- Black people’s weakest affinity to indicators was within the idealism (sense of justice, equality, constitution) and realism (survival, prosperity, group solidarity) categories.

### Interpretation of the results

Given that this was only a validation phase for the proposed measuring instrument, any interpretation of the results would be restricted to whether the measuring instrument is effective in its intended purpose. The following interpretations were made within the limitations noted above. Using the data from both the literature study and the empirical results within this context, it is clear that there is a disconnect between the expectations of the construct of national identity among the two categories of respondents which may explain the reasons for a declining construct among white people and a more pronounced construct among black people. As stated before, any doubts shown by members of the polity about the rationality of the system could weaken the legitimacy of the political identity and could inflict harm on the political system (Prinsloo 2016: 84; Xiaomei & Shimin 2014:162–163). It is probable that the strong emphasis by white people on idealist indicators of national identity show that their expectations have a historical basis, which is likely enshrined in South Africa’s Constitution. The declining national identity construct may be because of the perceived way in which South Africa’s ruling party, the African National Congress, has since the eradication of apartheid been moving away from the ideals enshrined in the constitution.

The results of the research further indicated that the construct of national identity is becoming comparatively stronger among black people. It is proposed that this does not necessarily imply that black people, especially the younger generation, is accepting the ruling party’s construct of national identity but rather that the social identification along ethnical lines are becoming more prominent. Evidently, for black people, the social identity construct, probably as a result of political affiliation and personal identification with the ruling political party, is the strongest. This means that the ruling party probably has a greater role to play among black people’s acceptance or construct of national identity than the state has (which enshrines the values of the constitution).

However, as was indicated from the literature study, the waning of expectations, especially among the youth, that the ANC, as the former liberation movement of South Africa, could continue to take the country to what is considered to be ‘genuine freedom’ and a new society (Bond 2014:20) may lead to a rejection of the ANC’s construct of national identity. Indeed, the formation of the national and political identity of South Africa remained stifled through a disconnection between the actions of the older generation who fought for freedom and the demands and expectations of the younger generation. Slowly but surely, the younger generation’s rejection of the current political elite has begun to weaken the legitimacy of South Africa’s political identity. Such a rejection could inflict real harm on the political system in South Africa (Prinsloo 2016:88).

Political risk, in the South African context, posed by violent service delivery protests, the widening income gaps, low levels of education, the lack of qualified work force, high crime and corruption levels and the high levels of unemployment have increased and remain some of South Africa’s most acute challenges, and this must be factored into in any discussion on South Africa’s future political landscape. One of the core dimensions of political risk is the national identity construct. The research clearly indicated that a declining national identity construct may weaken the state and increase political instability. In such an environment, it would not be possible to address developmental shortcomings and effectively apply measures to reduce disaster risk.

## Conclusion

With political risk defined as the potential negative consequences arising from political dynamics and social behaviour, and with South Africa being assessed with a medium risk profile, it should be noted that certain fault lines may exist along the different ethnic and age groupings in South Africa which could exacerbate its risk profile and potentially lead to a failing political system. With an ailing or eventually failing political system, it becomes unlikely that the aims of DRM to institute a continuous and integrated multi-sectoral, multidisciplinary process of planning and implementation of measures aimed at preventing or reducing the risk of disasters could be achieved. Successful DRM is thus dependent on a manageable political risk profile. Indeed, the ‘emerging cross-cutting’ interdisciplinary nature of DRM forces an expansion of its knowledge categories, especially in the study foci of government, governance and politics. Importantly for the vocationally orientated dimension of DRM, it becomes clear that a valuable connection exists between disaster risk and political risk: by expanding the study foci of government, governance and politics into foundational elements, such as political power relations, the dominant ideology of society and the construct of national identity, those elements of political risk which would increase disaster risk could be more thoroughly quantified and considered. From the literature, political risk and its comprising categories, DRM could be understood to have multiple compounding connections: firstly, higher political risk, in part measured by fissures in the national identity construct as well as its other elements, exacerbate disaster risk; secondly, higher political risk affects the capacity and the will of the government to effectively deal with disaster risk and employ disaster risk reduction measures.

By employing a tool to measure national identity, the measure of risk could qualitatively be incorporated into political risk and DRM. Of particular use was the determination of the different political ideologies within which the indicators of national identity fall. Not only does this aid to identify those indicators of national identity which are the least or most developed among interest groups, but it also allows comparing the different political ideological approaches that are seemingly important to the different ethnic groups. As a mitigating factor, this could point to possible contradicting expectations between ethnic groups to construct national identity and also the different approaches the state should consider to incorporate to successfully construct national identity. In summary, the research conducted and results provided in this article served to provide a possible approach to measure the level of development of the national identity construct in society and to qualitatively evaluate any inherent political risk and link it with disaster risk reduction.

## Footnotes

1 *Ubuntu* when interpreted as an ethical theory prescribes honouring relationships by sharing a way of life and caring for others’ quality of life (Metz 2014) (Prinsloo 2016).
